# Development of Gentamicin Bilosomes Laden *In Situ* Gel for Topical Ocular Delivery: Optimization, *In Vitro* Characterization, Toxicity, and Anti-microbial Evaluation

**DOI:** 10.34172/apb.2024.057

**Published:** 2024-07-31

**Authors:** Ameeduzzafar Zafar, Omar Awad Alsaidan, Malik Suliman Mohamed, Mohd Yasir, Mohammad Khalid

**Affiliations:** ^1^Department of Pharmaceutics, College of Pharmacy, Jouf University, Sakaka 72341, Al-Jouf, Saudi Arabia.; ^2^Department of Pharmacy, College of Health Sciences, Arsi University, Asella 396, Ethiopia.; ^3^Department of Pharmacognosy, College of Pharmacy, Prince Sattam Bin Abdulaziz University, Al-Kharj 11942, Saudi Arabia.

**Keywords:** Ocular delivery, In situ gel, Gentamicin, Bilosomes, ex-vivo permeation, Antimicrobial study

## Abstract

**Purpose:**

The eye drops are the prominent preparation used to treat surface eye disease (bacterial conjunctivitis). However, they have some limitations i.e., short corneal residence, resulting in low ocular bioavailability and necessitating frequent dose administration. The present study developed gentamicin (GE) bilosomes (BM)- laden *in situ* gel to improve therapeutic activity. The *in situ* gel system is initially in sol form before administration and converted into gel form in physiological eye conditions.

**Methods:**

The GE-BM was developed using the thin film hydration technique and optimized by D-optimal design. GE-BM was characterized for vesicle size, entrapment efficiency, zeta potential, morphology, and Fourier transform electron microscope (FTIR). The optimized GE-BM (GE-BMopt) was incorporated into an *in situ* gel and assessed for physicochemical characteristics. GE-BMopt *in situ* gel was evaluated for *in vitro* release, *ex vivo* permeation, toxicity, and antimicrobial study.

**Results:**

GE-BMopt has a vesicle size of 185.1±4.8nm, PDI of 0.254, zeta potential of 27.6 mV, entrapment efficiency of 81.86±1.29 %, and spherical morphology. The FTIR study presented no chemical interactions between GE and excipients. GE-BMopt *in situ* gel (GE-BMoptIG4) showed excellent viscosity, gelling strength, and *ex-vivo* bio-adhesion. GE-BMopt-IG4 showed significant high and sustained release of GE (78.08±4.73% in 12h). GE-BMopt-IG4 displayed 3.27-fold higher *ex-vivo* goat corneal permeation than a pure GE solution. GE-BMopt-IG4 showed good corneal tolerance without any damage or irritation. GE-BMopt-IG4 showed significantly (*P*<0.05) higher anti-bacterial activity (ZOI) against *Staphylococcus aureus *and* Escherichia coli* than pure GE solution.

**Conclusion:**

The study determined that the BM *in situ* gel system can serve as a substitute carrier for GE to enhance its therapeutic effectiveness, and further preclinical studies are required.

## Introduction

 The eye is one of the most dedicated organs of the body. Treating/managing the ocular disease (anterior or posterior) is a highly challenging task due to the complex anatomy and physiology of the eye. Conventional ocular topical preparations, such as eye drops, are effectively used for treating most surface diseases but can not stay for an extended period on the ocular tissue surface (cornea) due to defensive mechanisms of the eye (blinking, high tear fluid turnover).^1^

 Due to the low viscosity of eye drops, the majority of the administered dose (90%-95%) is eliminated from the eye surface, and some are drained by the nasolacrimal drainage canal and reach into systemic circulation, which may lead to systemic side effects.^2^ Quick elimination of the preparation from the eye surface, leads to a short duration of therapeutic effect. It was reported that long-time practice of eye drops may have harmful effects like inflammation in the cornea and conjunctiva, instability of tear film, and injury to the surface of the cornea.^3^

 There are various techniques reported to increase ocular bioavailability, i.e., using the viscosity enhancer^4^ and ointment,^5^ but these systems may produce blurred vision, burning, and itching in the eye.^6,7^ The other formulations such as the ocular insert,^8^ collagen shield,^6^ and implant^9^ system are reported to improve therapeutic activity and bioavailability. However, it has drawbacks such as difficulties in application and patient incompliance. Therefore, researchers have made a significant effort toward increasing the cornea contact time and permeation of the therapeutic agents in the eye for the treatment of ocular disease. Researchers have explored various drug-loaded nanoformulations for delivering medications to the eyes, with promising outcomes in terms of improving therapeutic efficacy. These formulations are Eudragit nanoparticles of terconazole,^10^ lactoferrin-encapsulated liposomes,^11^ latanoprost incorporated niosomes,^12^ ciprofloxacin-loaded bilosomes,^13^ solid lipid nanoparticles of indomethacin^14^ and itraconazole incorporated nanostructured lipid carrier.^15^ In that, the bilosomes (BM) are advanced and novel drug carriers for ocular delivery. It is a soft, flexible, and ultra-deformable nano vesicle composed of lipids, surfactants, and bile salt and it resembles conventional niosomes.^16^ Bile salt increases the membrane stability of the vesicle as well as enhances the permeation through the biological membrane.^17^ BM offers several advantages over conventional formulations in terms of protecting drugs from degradation and increasing drug stability.^16^ BM are capable of increasing ocular retention and permeation by adhering to the ocular surface. Furthermore, it enhances ocular bioavailability by decreasing drainage.^18^ There have been various studies reported on BM for ocular delivery. Abdelbary et al formulated the terconazole-loaded BM for ocular delivery and showed a significant (*P*< 0.05) higher permeation flux (3.30-fold) as compared to pure terconazole suspension.^19^ Mohsen et al formulated the acetazolamide-BM for ocular delivery and exhibited a higher percentage (40 %) of intraocular pressure reduction than pure acetazolamide (26%).^16^ Incorporation of BM into the hydrogel system (*in situ *gel) further increases the stability of BM and increases the corneal contact time or residence time of GM in the eye. The *in situ *gel is a *sol-to-gel* system, initially it is a solution form at non-physiological conditions and converted into gel after being instilled to eye surfaces (physiological condition pH-7.5, 37 °C).^20^ BM-laden *in situ *gel system provides high surface area, high corneal retention, prolonged drug release, maintained therapeutic concentration, and reduced dosing frequency. Janga et al formulated the natamycin-loaded BM *in situ *gel for ocular delivery and showed a 9-fold higher *ex vivo* corneal flux than plane natamycin suspension without any toxicity on the cornea.^21^ However, BM are good drug carriers, but their instability due to aggregation during long-term storage, and environmental susceptibility may pose challenges to scalability.^18,22,23^ Furthermore, there is very limited information available on their long-term safety profile in ocular tissues necessitating a thorough evaluation to ensure their suitability for therapeutic applications.^23,24^ The objective of this research was to develop of gentamycin-loaded bilosomes (GE-BM)-laden *in situ *gel that would enhance, ocular tolerance, and therapeutic efficacy. GE is an aminoglycoside antibiotic and can effectively combat a wide variety of Gram-negative and Gram-positive bacteria. However, its practical uses are often limited to modest dosage regimens due to the accumulation of residues in the kidney.^25^ GE has been applied topically as eye drops (solution) for the treatment of conjunctivitis and blepharitis. GE inhibited protein synthesis in bacteria by binding ribosome 30s unit.^26,27^ GE-BM was developed by the thin film hydration technique and optimized by D-optimal design using design expert software. GE-BM formulation was evaluated for vesicle size, PDI, zeta potential, entrapment efficiency, morphology, and interaction study. It has been hypothesized that incorporating GM into BM will increase its corneal permeability. Furthermore, because of their lipid bilayer structure, BM can adhere to the ocular surface, extending corneal contact time, sustaining ocular delivery, and improving therapeutic efficacy.^18^ Further, the optimized GM-BM formulation was incorporated intothe* in situ *gel using the stimulus-responsive gelling agents (Carbopol + HPMC). Then, the optimized GE-BM (GE-BMopt) *in situ *gel was evaluated for *in vitro* release, *ex-vivo* permeability, toxicity analysis, and antibacterial efficacy. Carbopol is a mucoadhesive characteristic and exhibits a sol-gel phenomenon as the medium pH rises over its pKa 5.5. HPMC acts as a viscosity-enhancing agent and augments the gel strength after application into the eye.^20,28^

 Until now, there are no reports available on GE-BM an *in situ *gel. There are various studies have been reported on GE-incorporated formulations for ocular delivery i.e., lipid-based micro-suspensions,^29^ GE-loaded Poly (lactic-co-glycolic acid) nanoparticles,^30^ chitosan nanoparticles laden hydrogel.^31^ In comparison to these prior reports, the present formulation is a promising carrier to improve the ocular delivery of therapeutic agents by combining the benefits of nano and *in situ gel* systems.

## Materials

 GE was procured from Unicure Pharmaceutical Ltd (Noida, India). Cholesterol, Span-60, and chromophore EL were supplied by Loba Chemie (Mumbai India). A dialysis bag (12 kDa) was procured from the Hi-Media (Mumbai, Maharashtra, India). HPLC-grade water, acetonitrile, and methanol were obtained from Loba Chemie (Colaba, Mumbai, India). All other chemicals utilized in this study were of analytical grade.

###  Experimental

####  Preliminary screening

 A thorough evaluation of existed literature survey was done for the selection of various formulation components and methods of preparation. Following existing literature review and preliminary studies were done to find the critical parameters that significantly affect the formulation characteristics, particularly vesicle size (VS, nm) and entrapment efficiency (EE, %). The bile salt (sodium glycocholate, ST), edge activator (Cremophor EL), surfactant (Span-60), cholesterol (CHO), and solvent system were selected based on major effects on VS and EE. The hydration time, hydrated solvent volume, and sonication time were taken from existing literature and subsequently verified through experimental trials.^32^ Different formulations were prepared to determine the range of independent variables in terms of quantity (mg) or percentage, as depicted in Table 1.

**Table 1:** Variables selected for the development and optimization of GE-BM by D-optimal design

**Independent variable**			**Dependent** **Variable**	**Goal**
**Name and unit**	**Level**			
	**Lower (-1)**	**Upper (+1)**		
Bile salt (sodium glycocholate, ST, mg)	10	30	VS (nm)	Minimum
Edge activator (Cremophor EL, % v/v)	0.25	0.75	EE (%)	Maximum
Surfactant (Span 60, (% w/v)	2	4	-	-

###  Preparation of GE-BM

 The GE-BM was prepared by following a previously published methodology with a few minor changes.^33,34^ The required amount of span-60, edge activator (Cremophor EL), CHO. and GE (20 mg) were taken into a round bottom flask (RBF) and dissolved into an organic solvent (chloroform: methanol, 1:1, 10ml). The flask was put on a rotary evaporator (BUCHI, Switzerland) at 45 °C under reduced pressure to evaporate the solvent. The thin film on the wall of RBF was formed and kept it in a desiccator overnight to eliminate the residual moisture. Then the thin film was hydrated by using bile salt (ST) solution on a rotary evaporator at 40 °C and 80 rpm. The GE-BM dispersion was formed and subjected to ultra-sonication (Ultrasonicator, Qsonica/USA) for 5 minutes at 30 seconds intervals (pulse mode). The GE-BM dispersion was collected in a glass vial, and stored at a cool temperature.

###  D-optimal design for optimization

 The D-optimal approach (Design Expert software®, version 8.0.6, State-Ease Inc., Minneapolis, USA) was used for optimization of GE-BM formulation.^35^ It is a form of computer-aided design that selects the most effective experiments from the total pool of possibilities. It helps to minimize the experimental run for the identification of optimized formulation and saves time and materials.^36^ The bile salt (ST, mg), edge activator (Cremophor EL, % v/v), and surfactant (Span 60, % w/v) were used as independent factors (low, medium, and high levels), whereas VS (nm, Y_1_) and EE (%, Y_2_) were taken as responses, respectively (Table 1). A total of 16 runs were obtained from the software to investigate the impact of the independent factors on the dependent factors (VS and EE) of the GE-BM (Table 2). Equation 1, which is a polynomial equation of the design model, illustrates the effect of the formulation component on the response (VS, EE).^37^

**Table 2:** Composition of GE-BM and data of Vesicle size and entrapment efficiency

**Formulation code**	**Bile salt (mg)**	**Edge activator (% v/v)**	**Surfactant (% w/v)**	**VS (nm)**		**EE (%)**	
				Practical*	**Predicted**	Practical*	**Predicted**
GE-BM-1	10.00	0.25	2.00	234.7 ± 7.2	239.03	71.53 ± 2.61	71.05
GE-BM-2	30.00	0.25	2.00	373.5 ± 11.7	382.02	52.47 ± 1.93	52.91
GE-BM-3	18.10	0.55	2.00	321.5 ± 9.1	326.77	67.72 ± 2.15	67.08
GE-BM-4	30.00	0.75	2.00	380.9 ± 5.6	381.83	52.91 ± 2.82	53.35
GE-BM-5	30.00	0.75	2.00	385.9 ± 9.4	381.83	53.91 ± 2.68	53.35
GE-BM-6	20.10	0.30	2.20	318.5 ± 12.4	297.68	62.23 ± 1.81	63.34
GE-BM-7	10.00	0.75	2.70	210.4 ± 6.5	205.69	74.5 ± 3.27	74.52
GE-BM-8	21.70	0.75	2.83	250.7 ± 7.8	259.13	63.13 ± 2.42	63.46
GE-BM-9	10.50	0.46	2.83	201.2 ± 5.9	204.30	73.42 ± 1.56	73.98
GE-BM-10	28.10	0.30	3.00	263.9 ± 7.5	262.73	64.62 ± 8	62.37
GE-BM-11	18.10	0.25	3.19	185.3 ± 4.3	189.62	70.21 ± 2.92	70.49
GE-BM-12	30.00	0.55	3.19	287.4 ± 7,8	284.30	59.94 ± 2.32	60.68
GE-BM-13	21.70	0.46	3.95	217.6 ± 5.3	223.31	75.32 ± 2.43	75.23
GE-BM-14	30.00	0.25	4.00	207.4 ± 6.8	205.94	74.63 ± 1.65	75.25
GE-BM-15	10.00	0.42	4.00	181.3 ± 4.6	178.29	81.42 ± 2.32	81.22
GE-BM-16	23.00	0.75	4.00	237.8 ± 8.9	235.52	69.64 ± 1.84	69.34

*Values are expressed as mean ± SD, n = 3.


(1) $$Y=\beta_{0}a_{0}+\sum \beta_{i}X_{i}+\sum \beta_{ii}X_{i}^{2}+\sum \sum \beta_{ij}X_{ij}$$

Where *β*_0_*, βi, βii,* and *βij*are the constant, linear, square, and interaction regression coefficients, and *Xi* and *Xj* indicate the independent variables. An analysis of variance (ANOVA) was done to examine the extent of the effect of independent factors on the responses. The regression value (R^2^) of all the applied models determined for selection of best fit model. The effect of independent factors on response, either alone or in conjunction with two factors of the best fit model, was explained by 3D and contour plots.^37^ The point prediction method of the software was used for the selection of GE-BMopt by further modification in the composition of the center point formulation. The different compositions of GE-BM formulations were prepared, and VS and EE were evaluated. The experimental data of all responses were compared with the software value and determined prediction error (equation 2). The desirability function value for the GE-BMopt was also examined.^38,39^


(2) $$Prediction\;error\left(\%\right)=\frac{Predicted\;value-Practical\;value}{Practical\;value}\times 100$$

###  Characterization of GE-BM

####  Vesicle size, PDI, and zeta potential evaluation

 The zeta sizer (Malvern, UK) instrument was used for the analysis of VS, PDI, and zeta potential of the formulation.^40^ The diluted GE-BM formulations (50 times) were filled into a quartz cuvette and placed in an instrument and analyzed the VS and PDI at 25°C. Zeta potential was measured by placing the sample into an electrode cuvette. The analysis was done at a 90° scattering angle and water was used as a dispersing medium (RI = 1.33).

###  Entrapment efficiency 

 The EE of the GE in GE-BM was determined using the ultracentrifugation technique.^16^ The GE-BM dispersions were filled into centrifugation tubes, and centrifuged at 19 000 rpm for 25 minutes (Remi-24 cooling centrifuge, Mumbai, India). The supernatant was collected and absorbance was determined by UV spectrophotometry (Genesys10S, Thermo Scientific, USA) at 257 nm after the required dilution.

###  Scanning electron microscopy (SEM)

 SEM instrument (SEM-HITACHI, Tokyo, Japan) was used to analyze the morphology of the GE-BMopt formulation. The sample was fixed over the adhesive–taped stubs, dried under a vacuum, and coated in a thin film (200 nm) with gold. Then it is placed into the instrument and captures the image.^41^

###  Fourier transform electron microscope (FTIR) 

 FTIR instrument (Shimadzu IRTracer-100, Japan) was used to examine the FTIR spectra of the GE, CHO, span-60, ST, and GE-BMopt. Each samples were mixed homogenously individually with potassium bromide and prepared pellet by a hydraulic pressure machine. Each samples were scanned at 4000-400 cm^-1^ at 25 ^º^C and capture the spectra.

###  Preparation of in situ gel 

 The pH-triggered methodwas used for the development of* in situ *gel formulation using carbopol 934P (gelling agent) and HPMC-K100 (viscosity enhancer) polymers.^28^ The specified quantities of carbopol 934P and HPMC-K100 (Table 3) were dissolved in an aqueous sodium chloride solution (0.9% w/v NaCl) and left overnight to ensure complete hydration. The GE-BMopt formulation (0.3% GE) was incorporated into the above polymeric solution and mixed properly to form a homogeneous preparation. Finally the preservative (0.02% benzalkonium chloride) was added and stored in glass vials at 25 °C until further evaluation.

**Table 3:** Various formulation compositions of GE-BEopt-in situ gel

**Formulation composition**	**Formulation code**				
	**GE-BMopt-IG1**	**GE-BMopt-IG2**	**GE-BMopt-IG13**	**GE-BMopt-IG4**	**GE-BMopt-IG5**
Carbopol 934P (% w/v)	0.5	0.1	0.2	0.3	0.4
HPMC E4M (% w/v)	0.25	0.25	0.25	0.25	0.25
Sodium chloride (g)	0.9	0.9	0.9	0.9	0.9
Benzalkonium chloride (%)	0.02	0.02	0.02	0.02	0.02
Distilled water (ml)	100	100	100	100	100

###  Characterization of GE-BM loaded in situ gel

####  pH, clarity measurement, and % transmission

 pH of the GE-BMopt *in situ *gel (GE-BMopt-IG) was examined by digital pH meter. The clarity of the formulation was measured under a white and black background.^42^ The % transmission of all GE-BMopt-IG was evaluated by a UV spectrophotometer at 480 nm using STF as a blank.

###  Viscosity determination

 Brookfield viscometer (V42000, Fungi Lab, Spain) was used to analyze the viscosity of all GE-BMopt-IG. The viscosity was measured at pH 5.4 and pH 7.4 (STF) for sol and gel form using spindle number 10S and 30 rpm.^42^

###  Gelling strength determination

 The 2 mL of STF (pH7.4, 2g NaHCO_3_, 6.7g NaCl, and 0.8 g CaCl_2_•2H_2_O in 1000 mL of water) was taken into glass vail separately and 2 drops of each GE-BMopt-IG formulation were added. Then observed visually and noted the gelling time as well as the retention time of the gels. Then, the gel was graded based on the formation of the gel and dissolution of the gel.^43^

###  Ex-vivo bio-adhesion study

 The physical balance method was employed to measure the bio-adhesion of the GE-BMopt-IG using the excised goat cornea. The bioadhesive strength was determined by detaching the cornea from the *in situ *gel.^44^ After the immediate scarification of the goat, the complete eyeball was isolated and stored in cool 0.9% NaCl. The cornea and sclera from the eyeball were carefully removed using a surgical blade and forceps. The cornea was tight to the opposite side of the balance pan. The GE-BMopt-IG formulation was placed into a petri dish and converted into a gel state by creating the physiological condition (STF, pH 7.4, 37 °C). The cornea was adhered to the gel intimately for 8min (preload time). Next, the weight was added to the second pan of the balance until the cornea became separated from the gel. The bio-adhesive strength was calculated by the given formula (dyne/cm^2^).


$$Bioadhesive\;strength=\frac{mg}{A}$$

m = weight (gram) applied for detaching the cornea, g = acceleration due to gravity, A = surface area of the cornea

###  In vitro drug release

 The *in vitro* drug release of GE from pure GE solution and GE-BMopt-IG was examined through dialysis bag mehod.^44,45^ The dialysis bag was dipped in distilled water overnight for the opening the pore. The dissolution medium (100 mL STF, pH7.4) was filled in the beaker and maintained at 37 °C on a thermostat magnetic stirrer. The 1 mLof formulations (0.3% of GE) were filled into a respected dialysis bag and tightly bound to both ends. Then it is immersed in a dissolution medium. The 3ml of the sample was taken at a predetermined time interval and the same volume of fresh STF was added to maintain diffusion. The aliquot was filtered through a syringe filter (0.45 µm) and the absorbance was examined by UV-spectrophotometry at 257 nm. The concentration was determined by the regression equation of the calibration curve and % release was calculated using Microsoft Excel. Various models were employed to assess the mechanism and kinetics of drug release by determining the regression coefficient (R^2^) and release exponent (n).^46^

###  Ex vivo goat corneal permeation study

 The diffusion cell (DHC-680, Logan Instrument USA) was used for analysis of permeation through the excised goat cornea. The excised cornea was mounted between the donor and acceptor parts of diffusion cell. The 10 mL of diffusion medium (STF, pH7.4) was filled into the acceptor compartment and maintained at 37 °C.^45^ The 1 mL of the pure GE solution and optimized GE-BMopt-IG was filled into the donor compartment. At a fixed time interval, the 1 mL aliquot was withdrawn from the diffusion medium and filtered by a 0.45 µm syringe filter. The GE content was determined by the previously validated HPLC method.^47^ HPLC instrument (Shimadzu LC10AD, Kyoto Japan) with a UV detector (254 nm) was used for the detection of GE. Acclaim120C18 column (4.6 mm internal diameter, 2.2 µm particle size) was used for separation. The mobile phase consists of 90:10% v/v acetonitrile and water. The flow rate was 0.75 mL/min. The volume of injection of the sample is 20 µL and the run time 20 minutes. The column temperature was 30 °C and the retention time was found to be 14.23 minutes.

###  Histopathological examination

 The excised goat cornea was submerged separately in an optimized GE-BMopt-IG and 0.9% NaCl solution for 6h and preserved in a 10% v/v formalin solution. Ethanol was used to dehydrate the cornea. A solid block was prepared by using paraffin wax and stained with hematoxylin and eosin dye. The histology image of the cornea was taken at 10 × 10x magnification using a Motic microscope (Motic, Japan).

###  Ocular hydration study

 The excised goat cornea was immersed in optimized GE-BMopt-IG for 24h. Then the cornea was removed and weighed (wet weight, D1). After that, the wet cornea was dried at 60 °C in a hot air oven for 72 hours and weighed again (dry weight, D2). The corneal hydration was calculated by the given equation.^48^


$$\%Corneal\;hydration=\frac{D1-D2}{D1}\times 100$$

###  In vitro irritation study

 HET-CAM (Hen’s Egg test-chorioallantoic membrane) method was used to evaluate the irritation potential of the GE-BMopt-IG.^49^ It is an *in vitro* test and an alternative to the Draize method for the determination of the irritative index.^50,45^ HET-CAM study assigns an irritation score to assess a substance’s propensity to irritate or damage the eye based on its effects on the CAM of a fertilized hen’s egg. The scoring was done on the basis of irritation potential such as hemorrhage, coagulation, and lysis of blood vessels of the CAM after formulation administration.^49^

 In the present study, the freshly fertilized hen eggs (not older than 7 days) were collected from the local poultry form and incubated in an incubator for 10 days at 37 °C and 51% RH. The eggs were rotated every day manually. At the end of the 10^th^ day, the eggs were removed from the incubator. The shells of the eggs were wisely removed from the air chamber side without any damage to the inner membrane. 0.9% NaCl solution was dropped over the inner membrane and removed properly without any damage to the CAM. Then eggs were distributed into three groups (group A: GE-MBopt-IG, group B: negative control 0.9% NaCl, group C: positive control 0.1M NaOH). The two drops of optimized GE-MBopt-IG, 0.9% NaCl, and 0.1M NaOH were added over CAM and the score of irritation was recoded visually for 0-5 minutes. The score was given on the basis of the irritating index *viz* 0-0.9 (non-irritating), 1-8.9 (irritating), and 9-21 (severe irritating).^45^

###  Sterility evaluation

 The sterility of the optimized GE-BMopt-IG was evaluated by using culture medium. The fluid thioglycollate (for bacteria) and soybean digested medium (for models) were prepared and sterilized at 121 °C. Then the optimized GE-BMopt-IG was inoculated into the growth medium and incubated for the growth of micro-organisms (14 days for fluid thioglycollate medium at 35 °C, and 5 days for soybean casein digest medium at 25 °C).^51^ Then observed visually under a white and black background for any turbidity, precipitation, etc.

###  Isotonicity evaluation

 The isotonicity of the optimized GE-BMopt-IG was evaluated using goat blood. The fresh blood was taken from the slaughterhouse into EDTA tubes. Then a drop of blood was mixed with optimized GE-BMopt-IG in a glass slide. The smear was made and stained with leishman stain. The image was captured by using the photomicroscope (Optical microscope, 40 × 40x).^52^

###  Antimicrobial study

 Antimicrobial evaluation of optimized GE-BMopt-IG and plan GE solution was done using the cup plate method. *Staphylococcus aureus* (RCMB 010010) and *Escherichia coli* (ATCC 8739) were used as test organisms. The nutrient agar medium was prepared and sterilized by the autoclave (CABN60801, Astell, England) at 121 °C. 15 mL of the sterilized nutrient agar medium was mixed with the strain into the petri dish and stood for solidification. The cup (4 mm) was made using a sterilized borer. The two drops of the optimized GE-BMopt-IG and plan GE (equivalent to 0.3% GE) were added into the respective cups and stood for 1 hour at 25 ºC. Then the plates were incubated in the incubator (Binder, USA) at 37 °C for 24 hours and the zone of inhibition (ZOI) was analyzed.

###  Statistical analysis

 GraphPad software (version 5, GraphPad, San Diego, CA, USA) was used for the statistical analysis. All data was expressed as mean ± SD. *P*< 0.05 to be considered for a significant effect. A one-way ANOVA along with a student T-test was used for the statistical analysis.

## Results and Discussion

###  Preliminary screening study for the selection formulation variables

Preliminary screening studies were carried out to improve our strategy and analyze the most influential factors in the fabrication of GM-BM.^18^ In the current study, three factors, *viz* bile salt (mg), edge activator (% v/v), and surfactant (% w/v) were dominantly affected on the responses (VS and EE) and selected from the preliminary study for the optimization by D-optimal design. Further, the outcomes of the preliminary screening showed that the vesicle could not develop at a low amount of CHO because it was unable to provide the requisite rigidity to the vesicle wall. On the other hand, a higher concentration of CHO produces a more rigid vesicular wall but exhibits a negative impact on the VS, EE, and drug release.^53,54^

###  D-optimal design

**Figure 1:**
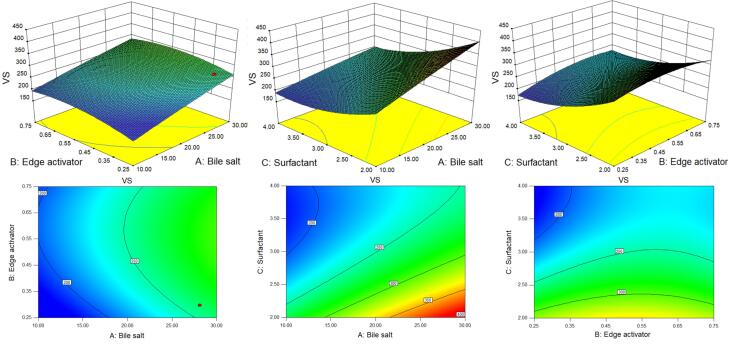
3D and contour plots showing the effect of different independent parameters on the vesicle size of GE-BM formulation

**Figure 2:**
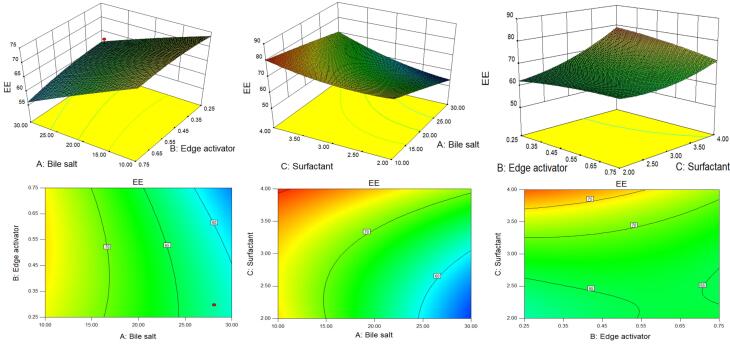
3D and contour plots showing the effect of different independent parameters on entrapment efficiency of GE-BM formulation

D-optimal design is a computer-aided design in which the best subset of all possible experiments is included.^55^ A selection process generates the best design possible by comparing it to a set of criteria across a certain number of repetitions of the design. The ability to use immethodical shapes and the availability of additional design points are potential benefits for optimization.^36^ The quadratic model and the lack of fit for VS and EE were found to be statistically significant (*P* < 0.05) and non-significant (*P* > 0.05) respectively. The high regression co-efficient value indicated the independent variables were a significantly (*P* < 0.05) effect on the responses (VS and EE). The individual and combined effects of the formulation variables on the VS and EE were shown by the 3D and contour plots (Figures 1 and 2) as well as by polynomial equations.

###  Effect of formulation factors on VS

 The polynomial equation (3) of the fitted model demonstrated how formulation factors affected the VS of the developed GE-BM.


(3) $$VS=+249.29+49.24X1+15.46X2-58.20X3-3.99X1X2-18.27X1X3+11.57X2X3-2.64X1^{2}-22.57X2^{2}+32.13X3^{2}$$

The quadratic model results demonstrate that all terms X1, X2, X3, X1X3, X2^2^, and X3^2^ are statistically significant (*P* < 0.05). The F-value of 65.27 suggests that the model is statistically significant (*P* < 0.05). The lack of fit for the best fitted was non significant (F = 11.80, *P* = 0.2173), and it is good for the model. The predicted R^2^ and adjusted R^2^ of the fitted model were 0.9899 and 0.9747, respectively. It displayed a strong alignment between the predicted and experimental values of the responses. The details of the best-fitted model and ANOVA of the VS is depicted in Tables 4 and 5 respectively. The adequate precision of 23.05 ( > 4) indicates a satisfactory signal-to-noise ratio.^37^ *P* < 0.05 is deemed to be significant for the model, whereas *P* > 0.05 is regarded as non-significant. Figure 1 shows the 3D surface response and contour plots of the fitted model, representing both individual and interactive effects of formulation variables on VS.

**Table 4:** Statistical summary of the applied model for Vesicle size and entrapment efficiency

	**Source**	**SD**	**R-Squared**	**Adjusted R** ^2^	**Predicted R** ^2^	**PRESS**	**CV**	**Selected model**
Vesicle size	Linear	25.98	0.8909	0.8636	0.7989	14916.20	-	-
	2FI	22.62	0.9379	0.8966	0.8281	12757.01	-	-
	Quadratic	11.18	0.9899	0.9747	0.8914	8054.20	4.20	Suggested
Entrapment efficiency	Linear	2.84	0.9155	0.8944	0.8360	187.60	-	-
	2FI	1.93	0.9707	0.9512	0.9083	104.94	-	-
	Quadratic	1.24	0.9919	0.9799	0.9178	94.01	1.86	Suggested

SD, standard deviation.

**Table 5:** ANOVA of the best-fitted quadratic model for Vesicle size and entrapment efficiency

**Source**	**Vesicle size**				**Entrapment efficiency**			
	**Sum of Squares**	**df**	**F value**	***P*** ** value Prob>F**	**Sum of Squares**	**df**	**F value**	***P*** ** value Prob>F**
Model	73440.23	9	65.27	< 0.0001*	1134.96	9	82.06	< 0.0001*
X1-Bile salt	17807.91	1	142.44	< 0.0001*	430.48	1	280.11	< 0.0001*
X2-Edge activator	1846.90	1	14.77	0.0085*	9.67	1	6.29	0.0460*
X3-Surfactant	24877.81	1	199.00	< 0.0001*	231.33	1	150.52	< 0.0001*
X1X2	78.58	1	0.63	0.4581**	9.74	1	6.34	0.0454*
X1X3	1541.83	1	12.33	0.0126*	36.62	1	23.83	0.0028*
X2X3	661.17	1	5.29	0.0611**	37.10	1	24.14	0.0027*
X1^2^	19.58	1	0.16	0.7060**	0.21	1	0.14	0.7258**
X2^2^	1243.64	1	9.95	0.0197*	1.99	1	1.30	0.2982**
X3^2^	2903.35	1	23.22	0.0029*	23.28	1	15.15	0.0081*
Residual	750.10	6			9.22	6	-	-
Lack of Fit	737.60	5	11.80	0.2173*	8.72	5	3.49	0.3847**
Pure Error	12.50	1	-	-	0.50	1	-	-
Cor Total	74190.33	15	-	-	1144.18	15	-	-

*P < 0.05 = Significant, **P > 0.05 = Non-significant.

The positive and negative signs of the polynomial equation showed the synergistic and antagonistic effects of variables over the VS of GE-BM. The VS of all formulations ranged from 181.3 ± 4.6 (GE-BM-15) to 385.9 ± 9.4 nm (GE-BM-5) (Table 2). Bile salt (coefficient + 49.24) and edge activator (coefficient + 15.46) exhibited a positive impact, but surfactant (coefficient -58.20) displayed a negative impact on VS. The VS of GE-BM increases by increasing the ST (X1) concentration because of the high interaction between ST and lipid and altered lipid packing, hence increasing the VS of GE-BM. Furthermore, the bulkiness effect of ST would cause an increase in VS.^56^ These results are in agreement with the previously reported finding, i.e., betaxolol hydrochloride-BM and showed enhanced ocular permeability to combat glaucoma.^39^ The second factor Cremophor EL (X2) showed a positive effect on the VS of GE-BM. It is due to the development of a bulky shield and steric stabilization.^57^ Cremophor EL contains both hydrophilic (polyethylene oxide, PEO) and hydrophobic moieties.^19^ The abundance of hydrophilic PEO residues, specifically three PEO chains in Cremophor EL (total 35 PEO units), resulted in enhanced water absorption and subsequent increase in the VS.^19,54^ Similar type of observations were reported by Abdelbary et al in terconazole BM for ocular delivery.^19^ Surfactant (Span 60) showed a negative and dominant effect on the VS than other factors. The VS increased with increasing the span-60 concentration due to the high organization packing of molecules in the BM layer. In addition, it increases the stability of BM, which prevents the aggregation of vesicles. However, with an increase in the span-60 concentration, the interfacial tension between the CHO and aqueous phase was decreased, thereby leading to a reduction in the VS as well as minimizing the coalescence. The same type of finding was stated in acyclovir-loaded BM for oral delivery.^58^

###  Effect of formulation variables on the EE

 The polynomial equation (4) of the fitted model demonstrated how formulation factors affected the EE of GE in developed GE-BM.


(4) $$EE=+67.51-7.66X1-1.12X2+5.61X3-1.40X1X2+2.82X1X3-2.74X2X3-0.27X2^{2}+0.90X2^{2}+2.88X3^{2}$$

The quadratic model is the best-fitted model (*P* < 0.0001, F-value = 82.06, R^2^ = 0.9919) compared to other models (Table 4). The quadratic model terms X1, X2, X3, X1X3, X1X3, X2X3, and X3^2^ are statistically significant (*P* < 0.05) on EE of GE in GE-BM (Table 5). The lack of fit was found to be non-significant (F = 3.49, *P* = 0.3847) as compared to the pure error. The R^2^ and adjusted R^2^ of the fitted model are 0.9919 and 0.9799, showing a strong alignment between the regression model and the experimental data. The adequate precision is 28.88 ( < 4) indicates a satisfactory signal-to-noise ratio.^37^

The details of all models and the analysis of variance of best fit model for EE are depicted in Tables 4 and 5 respectively. Figure 2 shows the 3D surface response and contour plots, demonstrating both the individual and interactive effects of the formulation variables on EE. The positive and negative signs in the polynomial equation displayed the synergistic and antagonistic effects on the EE of GE in GE-BM. The EE of GE in all GE-BM was found to be in the range of 52.47 ± 1.93% (GE-BM2)- 81.42 ± 2.32% (GE-BM15) (Table 2). The ST (X1, coefficient -7.66) and cremophor EL (X2, coefficient -1.12) exhibited a negative impact on the EE of GE in GE-BM, but the surfactant (coefficient + 5.61) displayed a positive effect. An increase in the concentration of ST (X1) led to a decrease in the EE of GE in GE-BM. This phenomenon could be attributed to the development of micelles in the dispersion medium, which increases the solubility of GE in the external/dispersion phase and consequently decreases EE. Furthermore, an increase in ST content resulted in a fluidizing effect on the lipid bilayer, which led to a drop in EE because of drug leaked into the external medium. Cremophor EL displayed an opposite effect on the EE of GE in GE-BM, but was less prominent than ST.^19,53,59^ An increased amount of cremophor EL, results in the formation of more pores inside the vesicular structure, which will make the structure more permeable.^19^ In addition, an increase in the cremophor EL concentration may increase the fluidity of the bilayer, which results in a reduction of EE.^60^ Moreover, the cremophor EL (edge activator) might behave like bile salt and may enhance the solubility of the drug into the external phase, and the formation of possible mixed micelles may take place, thereby decreasing the EE.^19^ The third factor surfactant (span 60) displayed a positive impact on EE. The EE of GE in GE-BM increased as the concentration of span 60 increased.^61^ The elevated transition temperature and long alkyl chain of span 60 contributed to achieving a greater EE of GE in GE-BM.^53^ Similar findings were stated in zolmitriptan-loaded BM nose-to-brain delivery.^62^

###  Adequacy check of the model and selection of optimized formulation

**Figure 3:**
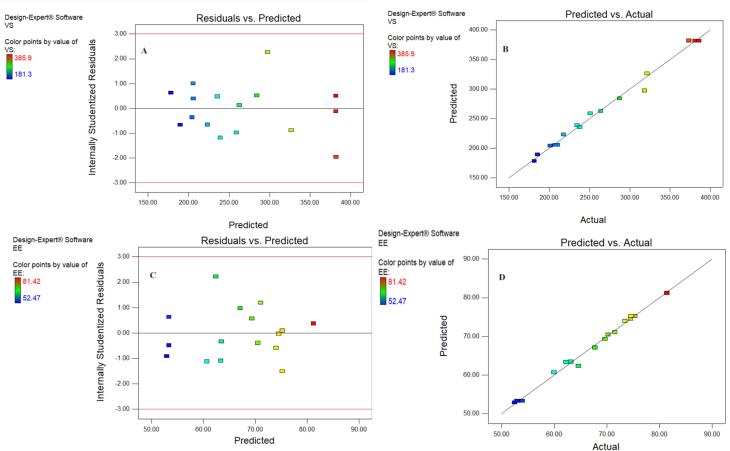
The studentized residuals & the predicted value graph (A) actual & predicted value graph (B) of vesicle size and studentized residuals & the predicted value graph (C) actual & predicted value graph (D) of entrapment efficiency of GE-BM formulation

An adequacy check study is necessary to assess the data analysis of the model, ensuring its accuracy in representing the real system. Failure to do so may result in poor or misleading findings.^37^ Figures 3A and 3B display the plot of the studentized residuals against the VS of GE-BM. This plot displays a random scattering of the observations of the response indicating that the variance remains constant across all response values. Similar findings were observed by Abbad and co-workers in ZnO nanoparticles optimization by D-optimal design.^37^ Similar the adequacy check study results were observed for EE, as depicted in Figures 3C and 3D.

**Figure 4:**
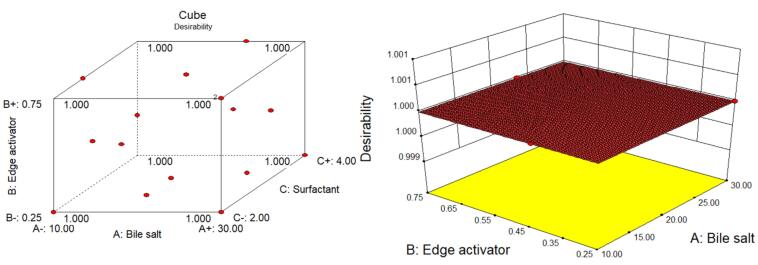
Optimization by D-optimal design showing the desirability graphs: Cube (A) and 3D diagram (B) of optimized GE-BM formulation

The desirability function test was applied to assess the accuracy of the model and identify the optimal composition of independent variables to get the optimum VS and maximum EE of GE in GE-BM. The optimized composition for GE-BM was selected from point prediction of the software. The GE-BMopt has 14 mg of bile salt (ST), 0.4 % v/v of edge activator (cremophor EL), and 4 % w/v of surfactant (span-60). The predicted value of the response is 188.3 nm of VS and 79.85% of EE. However, the actual value of responses is 185.1 ± 4.8nm of VS and 81.86 ± 1.29nm of EE. The prediction error was calculated and found to be -1.73 for VS and 2.46 for EE. There is a low percentage of prediction error, justifying the validity of the response. The desirability function value of the GE-BMopt was found to be 1, revealing the robustness of the design (Figure 4A-B). By visualizing multiple responses simultaneously, the 3D desirability graph (Figure 4B) allows for the identification of optimal formulations that meet all desired criteria and trade-offs among different variables. A cube desirability graph is an extension of the 3D desirability graph, typically used to optimize of formulations with more than three variables.^55^ These figures indicate that the findings of responses were aligned with the predicted values. It indicates that D-optimal design with the desirability function is a favorable method for the optimization of the formulation. Similar types of findings were reported in the optimization of topical microemulsions of itraconazole using a D-optimal design.^63^

###  Evaluation of optimized GE-BMopt formulation

###  Vesicle size, PDI, and zeta potential

**Figure 5:**
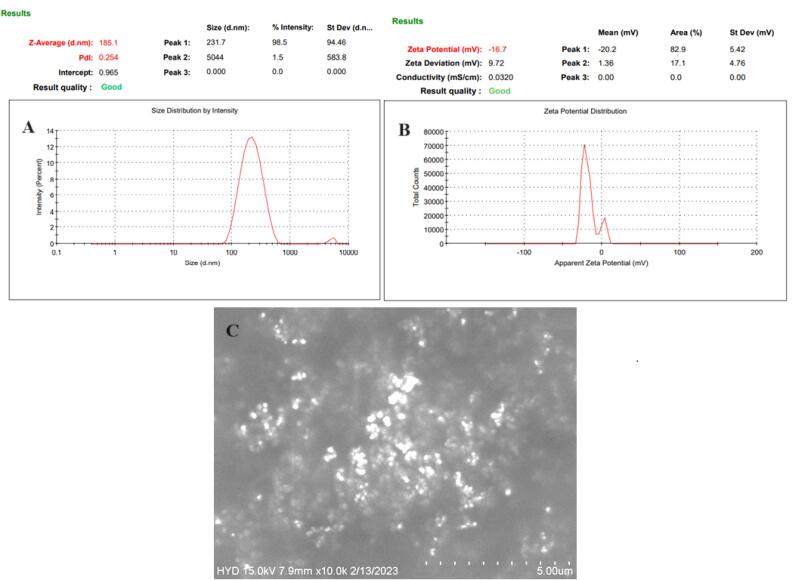
Expresses the vesicle size (A), Zeta potential (B), and SEM image (C) of the GEBMopt

The VS and PDI of GE-BMopt were analyzed and found to be 185.1 ± 4.8 (Figure 5A) and 0.254, respectively. The DPI is < 0.5 indicating the uniformity of VS distribution. The particles in range 50-400 nm are considered for ocular delivery and can pass the ocular barriers without any irritation.^64^ Zeta potential is the charge on the particle, which define the stability of formulation. The zeta potential of GE-BEopt was -27.6 mV (Figure 5B), indicating the formulation had sufficient charge and was capable of preventing particle aggregation.^65^

###  Entrapment efficiency 

The EE of GE in all GE-BM formulations was analyzed and found to be in the range of 52.47 ± 1.93% (GE-BM-2) - 81.42 ± 2.32% (GE-BM-15) respectively. The EE of the GE in GE-BEopt was 81.86 ± 1.29 % (Table 6). Sufficient entrapment of drug in formulation is necessary to ensure the concentration of the drug at the site of action.^64^

**Table 6:** *In vitro* characterization parameters of developed *in situ* gel

**Code**	**Clarity**	**% Transmission**	**Viscosity**		**Gelling strength**		**Drug content**
			**Sol state ** **(pH5.4±0.2)**	**Gel state ** **(STF, pH 7.4±0.2)**	**Sol ** **(pH5.4±0.2)**	**Gel ** **(STF, pH 7.4±0.2)**	
GE-BMopt-IG1	Transparent	97.53	13.42	45.12	--	-	96.65 ± 2.75
GE-BMopt-IG2	Transparent	96.32	28.53	165.43	-	+	97.43 ± 1.92
GE-BMopt-IG3	Transparent	95.25	66.76	246.71	-	+ +	97.91 ± 2.03
GE-BMopt-IG4	Transparent	87.98	105.34	387.23	-	+ + +	99.16 ± 1.02
GE-BMopt-IG5	Translucent	80.43	187.92	687.54	+	+ + + +	99.72 ± 0.48

(-) No gel formation, ( + ) gel formed in 1min and dissolved within 30min, ( + + ) gel for in few seconds and dissolved in 2 h, ( + + + ) gel formed quickly and stayed > 24 h, ( + + + + ) gel formed very quickly and stayed for > 24 h (very hard gel).

###  Morphological examination

The SEM instrument was used to examine the morphology of the GE-BMopt and the image shown in Figure 5C. The image displayed the vesicles are spherical with smooth surfaces. The spherical shape of the vesicle participated in improving biocompatibility, permeation across ocular barriers, and reducing irritation, as well as maintaining drug stability.^16^

###  Fourier transform electron microscope 

**Figure 6:**
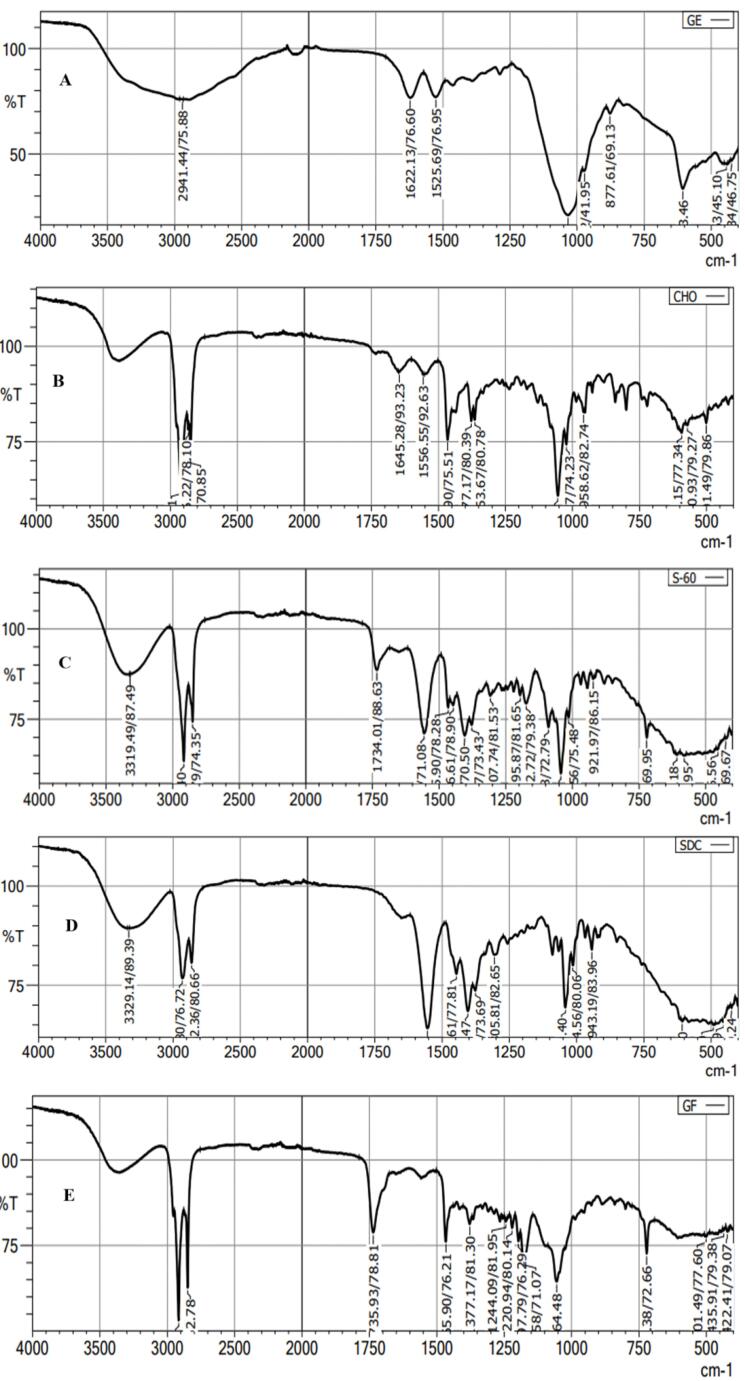
FTIR spectra of pure GE (A), Cholesterol (B), Span-60 (C), Sodium glycocholate (D), and GE-BMopt (E)

The FTIR spectra of GE, CHO, span-60, ST, and GE-BMopt were analyzed, and the spectra are shown in Figure 6. The FTIR spectra of GE showed (Figure 6A) the characteristic vibrational stretching band at 3390cm^-1^ (N-H stretching), and 2941cm^-1^ (C-H stretching). The other vibrational bands at 1622 cm^-1^ (N-H bending of aromatic ring), and 1525 cm^-1^ (N-H bending of aromatic ring)) were also recorded confirming the genuinity of GE. The spectra of CHO showed the characteristic vibrational peaks at 3440 cm^-1^ (O-H stretching), 2918 cm^-1^, 2866 cm^-1^ (C-H stretching), 1465cm^-1^, 1377 cm^-1^ (C-H bending), confirming the purity of cholesterol (Figure 6B). The span-60 showed (Figure 6C) the vibrational band at 2916-2850 cm^-1^ (aliphatic symmetric and asymmetric stretching), 1734cm^-1^ (C = O stretching) 1195cm^-1^ (-C-CO-O stretching). The spectra of ST expressed their own characteristics peaks at 3329 cm^-1^, 2931-2862 cm^-1^ (C-H symmetric and asymmetric stretching), and 1554 cm^-1^ (O-C = O stretching), respectively (Figure 6D). However, characteristic peaks of GE are present in the FTIR spectra of GE-BMopt revealing no chemical interaction between the drug and excipients (Figure 6E). Similar types of results were shown in doxorubicin-loaded liposomes^66^ and diclofenac Sodium-loaded BM.^67^

###  Development of GE-BMopt-laden in situ gel

The *in situ *gel system of GE-BM was successfully prepared by using carbopol 934P (gelling agent) and HPMC-K100 (viscosity enhancer) (Table 3) and evaluated.

###  pH, clarity, and % transmission

The pH of the GE-BMopt-IG was found to be 5.4 ± 0.2 in the sol state. However, pH at gel state was 7.4 ± 0.2 (in STF), indicating that the GE-BM *in situ* gel formulations were compatible with the eye. All the GE-BMopt-IG formulations were found to be transparent except GE-BMopt-IG5 because it contains a high concentration of the gelling agent (Table 6). The % transmission of all GE-BMopt-IG was found to be 97.53-80.43 % (Table 6). The developed formulation exhibited acceptable pH, clarity, and transmission to guarantee patient safety, comfort, and therapeutic effectiveness.^19,42^

###  Viscosity determination

Sufficient viscosity of the *in situ *system is required for conversion into a gel state on the contact of STF and stays for a longer time on the cornea, preventing rapid clearance and releasing the appropriate amount of drug for therapeutic action in a sustained manner. The viscosity of All the GE-BMopt-IG systems was determined in sol and gel forms, and the result was given in Table 6. The viscosity of all formulations in sol form was found to be in the range of 15.92 (GE-BMopt-IG1) to 187.92 (GE-BMopt-IG5). It showed that the increasing concentration of polymers the viscosity of the *in situ *gel increased because of crosslinking between polymers and formation of a high network structure.^68^ It directly influenced the gelling strength of the *in situ *gel and the release of the GE from GE-BMopt-IG. The *in situ *gel exhibited pseudo-plastic under physiological conditions. Similar types of observations were reported in the *in situ *gel system of dorzolamide for ocular delivery.^69^

###  Gelling strength

The gelling strength of the *in situ *gel is very important because it directly influences the retention of formulation in the cul-de-sac of the eye and the sustained release of the drug from the *in situ *gel. The gelling strength ofall GE-BMopt-IG formulations was determined, and the results are given in Table 6. The order of gelling strength of all formulations isGE-BMopt-IG1 < GE-BMopt-IG2 < GE-BMopt-IG3 < GE-BMopt-IG4 < GE-BMopt-IG5. The signs, i.e., (-) no gel formation, ( + ) gel formed in 1 min and dissolved within 30 min, ( + + ) gel for a few seconds and dissolved in 2 hours, ( + + + ) gel formed quickly and stayed > 24 hours, ( + + + + ) gel formed very quickly and stayed for > 24 hours (very hard gel). The gelling strength increases with increasing the concentration of gelling polymer because it increases the viscosity of the sol system. This is due to the pH-sensitive carbopol gelling polymer.^52,70^ It is converted into gel form when the pH of the sol state > 5.5. It revealed that use of combination of polymers would fulfill the criteria for the development of an appropriate gel in a short gelation time in STF. Thus, such preparation will increase the ocular residence time and sustained release of the drug after installation and overcome the limitations of topical conventional eye drops. A similar type of observations was recorded in betaxolol niosomes *in situ *gel,^71^ and moxifloxacin incorporated *in situ *gel^72^ on contact with STF.

###  Drug content 

GE content in all *in situ *gel formulations was analyzed and found to be 96.65 ± 2.75% (GE-BMopt-IG1) to 99.72 ± 0.48 (GE-BMopt-IG5). The optimized *in situ *gel (GE-BMopt-IG4) has 99.16 ± 1.02% GE content (0.3% of GE). Based on the above characterization parameters of the evaluation of the *in situ *gel system, the GE-BMopt-IG4 was selected as the optimized formulation, and the result of all parameters is expressed in Table 6. GE-BMopt-IG4 has good consistency and optimum gelling strength.

###  Ex-vivo bio-adhesion study

The bio-adhesive study of the GE-BMopt-IG4 was measured and found to be 753.84 dyne/cm^2^. The bio-adhesion of the GE-BMopt-IG4 was found to be 5.03-fold higher than the shear stress of the corneal film (150 dyne/cm^2^). The significant (*P* < 0.05) high bio-adhesion directly influenced the formulation’s residence time on the cornea (increases the time), and was not easily cleared by tear fluid turnover, blinking, or other protective mechanisms of the eye. Ranch et al developed the *in situ* gel with carbopol and showed excellent bioadhesive potential.^52^ Another study of an *in situ *gel formulation with carbopol and HPMC polymers exhibited excellent bio-adhesion over the excised mucin of the cornea due to hydrogen bonding.^73^

## *In vitro* drug release

**Figure 7:**
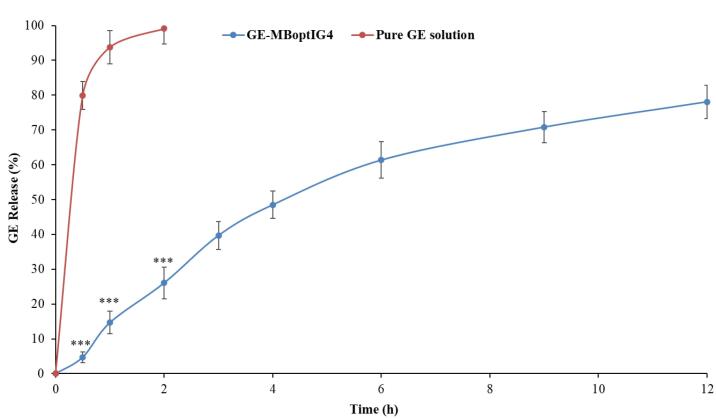
In-vitro GR release profile from GE-MBopt-IG4 formulation and pure GE solution. Values are given as SD±mean, n=3, Comparison of % GE release from GE-MBopt-IG4 was made with pure GE solution at ***P<0.001. *** indicates the difference is highly significant.

Figure 7 shows the release of GE from the pure GE solution and GE-BMopt-IG4 in STF at 37 °C. The release of GE was found to be 99.09 ± 4.41% (2h) from pure GE solution and 78.08 ± 4.73 % (12h) from GE-BMopt-IG4. The pure GE solution releases about all GE in 2h because of its aqueous solubility. However, the GE-BMopt-IG4 exhibited a dual release profile of GE, initially fast release, i.e., 26.07 ± 4.52% (2h) and later extended releases (sustained release, i.e., 78.08 ± 4.73 % (12h). The fast release may be due to the surface-deposited release of GE from the BM. However, the later slow and sustained release is due to the release of encapsulated GE from GE-BMopt-IG4. In addition, the gel also provided an additional barrier and gave a sustained release profile, because carbopol converts into gel at above pH 5.5. The result indicated that the *in situ *gel system increases the corneal contact time, prevents the wastage of the drug, and decreases the dosing frequency. Rarokar et al formulated the terbinafin-lipid-based nanoparticles and converted them into an *in situ *gel for ocular delivery. It exhibited 95.47 % terbinafin release in 24h.^74^ Hosny et al formulated the nano-lipid laden *in situ *gel of natamycin for topical ophthalmic application. They reported about 100% of drug release in 24h as compared to pure drug (100% in 4h).^75^ Allam et al developed niosomes loaded with betaxolol-loaded in situ gel using carbopol and HPMC for ocular delivery and showed sustained release of the betaxolol.^71^ The Higuchi model was found to be best fitted (R^2^ = 0.9771). The release exponent (n) was 0.85, indicating the release of drug through non-Fickian diffusion mechanism.^46^

## *Ex vivo* permeation

**Figure 8:**
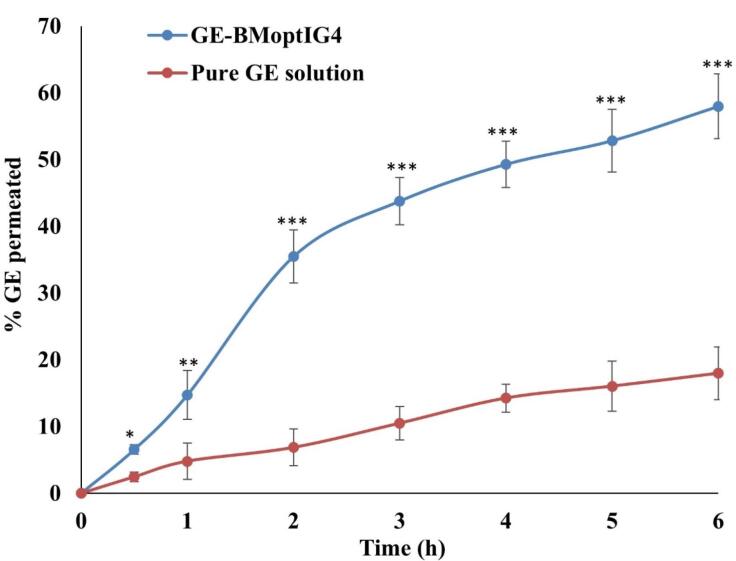
Ex-vivo goat permeation of GE from GE-BM-opt-IG4 and pure GE solution. Values are given as SD ± mean, n = 3, Comparison of % drug permeated of GE-MBopt-IG4 was made with pure GE solution at *P < 0.05,**P < 0.01, and ***P < 0.001. * OR** indicates the difference is significant and *** indicates the difference is highly significant

*Ex vivo* goat corneal permeation of GE from the pure GE solution and GE-BMopt-IG4 was analyzed using the diffusion cell, and the results are shown in Figure 8. The % permeation of GE from the pure GE solution and GE-BMopt-IG4 was found to be 17.97 ± 3.96% (53.94 ± 11.90 µg) and 57.97 ± 4.84 % (173.91 ± 14.53 µg) respectively. The flux was calculated using the regression equation of the plot and found to be 46.80 ± 3.13 µg/cm^2^.h for GE-BMopt-IG4 14.28 ± 2.14 µg/cm^2^.h respectability. The APC of GE from GE-BMopt-IG4 showed 3.16 fold higher (2.6 × 10^-3^ cm^2^/min) than pure GE solution (8.22 × 10^-4^ cm^2^/min). The higher permeation of GE from GE-BEopt-IG4 is due to the presence of surfactant and bile salt in the formulation. The bile salt can fluidize the biological membrane lipid (cornea) and increase permeation.^19^ In addition, bile salt enhanced the flexibility of BM vesicles and allowed the permeation of vesicles across the cornea.^76^ The high permeation is also due to the nanosize of the vesicles, which easily enter to corneal membrane through the endocytosis mechanism. The bioadhesive and permeation enhancing characteristics of carbopol, as well as it significant gelling power, which increases the corneal contact time, prevents the loss of drug and increased the permeation.^77-79^

###  Corneal hydration

 This study was done to determine the hydration state because it directly affects the thickness microstructure, and mechanical characteristics of the cornea.^80^ It also showed the tolerance capacity of the cornea. The hydration of the cornea after treatment with GE-BEopt-IG4 was found to be77.03 ± 0.82%. The corneal hydration was found to be under the limit as reported in the literature (76-80%)^81^ and revealed that GE-BEopt-IG4 did not produce any irritation or injury and maintained physiological hydration. The same type of finding was stated in the flurbiprofen nano-vesicle for ocular delivery.^82^

###  Histopathology

**Figure 9:**
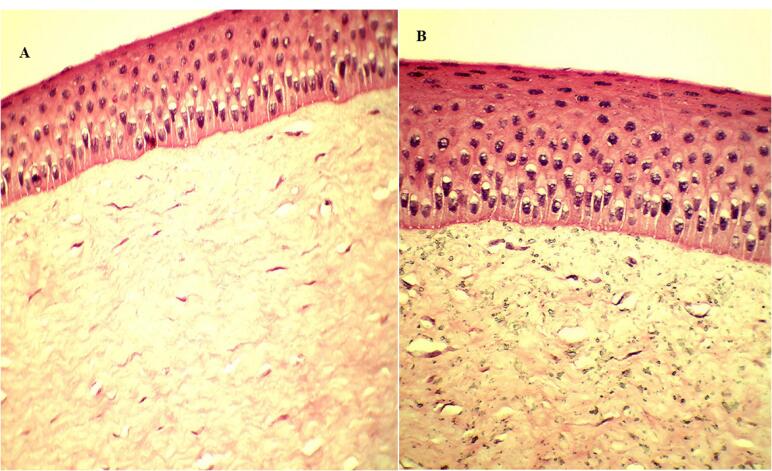
Histopathology of excise goat cornea after being treated with GE-BMopt-IG4 formulation (A) and 0.9% NaCl solution (B)

 Histopathological study of excised goat cornea was done to examine the corneal damage after treatment with GE-BMopt-IG4 and the result is depicted in Figure 9. It was observed that theGE-BMopt-IG4 did not damage (irritate) the cornea (Figure 9A) and showed similar findings to the normal saline treatment cornea (Figure 9B). It revealed that the ingredient used for the development of GE-BM loaded *in situ *gel is safe for ocular delivery. Similar findings were reported by Dahiya et al in forskolin and rutin-loaded polymeric nanoparticles for ocular delivery.^45^

###  HET-CAM

 HET-CAM study was done to quantitatively evaluate the degree of irritation caused by the GE-BMopt-IG4 and compare it with the control (standard).^50^ GE-BMopt-IG4 showed 0.2 scores (0-0.9 score nonirritant), revealing that it did not damage the CAM of hen eggs. In addition, 0.9% NaCl did not show any irritation of CAM (0 score), while the 0.1M sodium hydroxide produced the potential irritation (severe damage) to CAM (16.2 score, 9-21 severe irritant).^50,83^ These results were agreed to ketoconazole nanoemulsion *in situ* gel for ocular delivery.^84^

###  Isotonicity evaluation 

**Figure 10:**
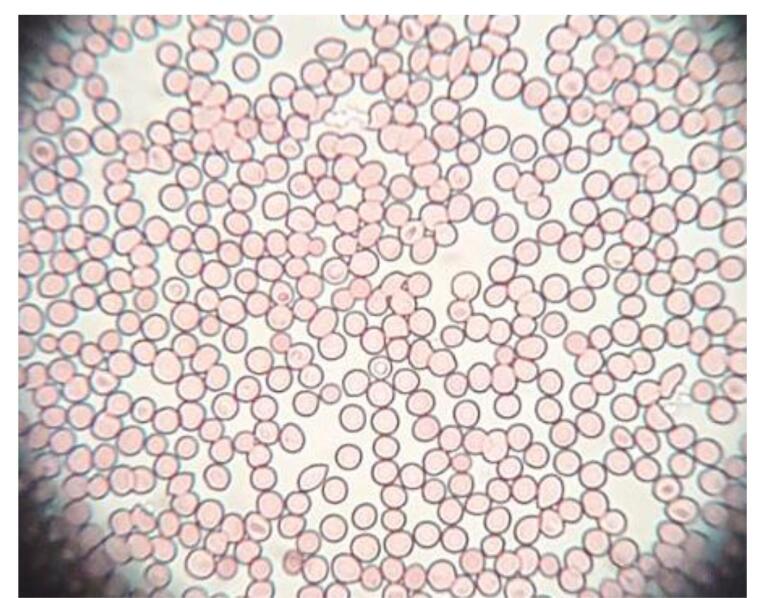
Showing the image of RBC of goat blood after treated with GE-BMopt-IG4 formulation (no shrinking, no swelling)

 The isotonicity study of GE-BMopt-IG4 sample was done using fresh goat blood, and the result is depicted in Figure 10. The GE-BMopt-IG4 did not show any swelling, shrinking, or breaking of red blood cells after treatment. It revealed that the formulation was non-irritant and isotonic.^64^

###  Sterility test 

 The sterility of GE-BMopt-IG4 was evaluated using fluid thioglycollate and soybean digest medium. After incubation at a definite period, it did not show any turbidity or precipitation in the medium and revealed that the formulation was sterilized.

###  Antimicrobial study

**Figure 11:**
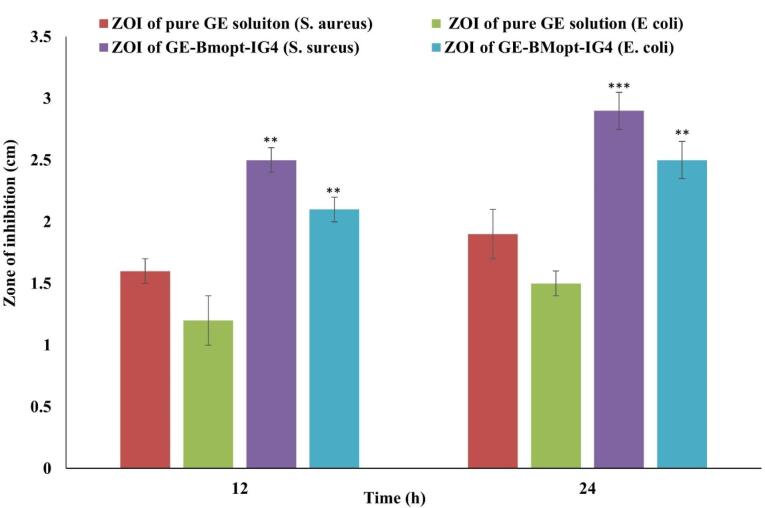
Antimicrobial study of pure GE solution and GE-BMopt-IG4 on stains *(S. aureus, E. coli*). Values are given as SD ± mean, n = 3, Comparison of antimicrobial activity of GE-MBopt-IG4 was made with pure GE solution against *S. aureus, and E. coli* at **P < 0.01, and ***P < 0.001. ** indicates the difference is significant and *** indicates the difference is highly significant

*Staphylococcus aureus* (gram-positive) and *E. coli *(gram-negative) are commonly used test organisms in evaluating ocular drug delivery systems due to their relevance to ocular infections and standardized testing (widely accepted for antimicrobial susceptibility testing and efficacy evaluations). Researchers can assess the broad-spectrum antibacterial activity, accessibility, and handling safety using normal microbiological methods by using both gram-positive and gram-negative bacteria.^85,86^ In the present study, the antimicrobial potential activity of GE-BEopt-IG4 and pure GE solution was assessed on *S. aureus* and *E. coli *using the cup plate method. Figure 11 displays the antibacterial activity of GE in both formulations. The GE-BMopt-IG4 showed the ZOI against *S. aureus* is 2.5 ± 0.1cm in 12h and 2.9 ± 0.15cm in 24h respectively. GE-BMopt-IG4 showed the ZOI against *E. coli *is 2.1 ± 0.1cm in 12h and 2.5 ± 0.1cm in 24h respectively. However, the pure GE solution exhibited ZOI 1.6 ± 0.1cm and 1.9 ± 0.2cm against *S. aureus *and 1.2 ± 0.2cm and 1.5 ± 0.2cm against *E. coli* respectively. The GE showed significantly higher activity against *S. aureus *than* E. coli*. GE-BMopt-IG4 showed higher antimicrobial activity against both tested micro-organisms at all times point. The GE kills bacteria by inhibiting protein synthesis by binding to the 30S ribosome. The high activity of GE in GE-BMopt-IG4 is due to the nanosize of vesicles and high flexibility BE, which may enhance the membrane permeability.

## Limitations and futuristic scope of the study

 The study focused on ocular bacterial infection using GE-BM, but we did not compare the standard treatment for ocular infection. Comparative studies with standard treatments would provide a better understanding of the formulation’s effectiveness and potential advantages, limiting the generalizability of the findings in our case. Although preclinical research yielded encouraging findings, further safety and effectiveness assessments in human subjects are necessary and may have future scope before translating GE-BM into clinical practice. Even though bilosomes are nontoxic formulations, ensuring they do not induce toxic effects on ocular tissues is essential for their clinical application. Proper stability of bilosomal gel must be ensured, because it is sensitive to environmental factors like temperature, pH, and mechanical stress. Some other formulations, like surface-modified bilosomes and lipid nanoparticles, may be developed in the future with better ocular application features.

## Conclusion

 The GE-BM was successfully prepared and optimized by D-optimal experimental design by using span-60 (surfactant), chromophore EL (edge activation), ST (bile salt) and CHO. The GE-BMopt showed nanosize vesicle (185.1 ± 4.8nm), high zeta potential (-27.6mV), and high % entrapment of GE (81.86 ± 1.29%). The shape of GE-BMopt vesicle was found to be spherical. The GE-BMopt formulation was successfully incorporated into an *in situ *gel system using HPMC-K4M (viscosity enhancing) and carbopol 934P (gelling agent) polymers. The GE-BMopt-IG4 has a transparent appearance, excellent viscosity (387.23cps) in the physiological environment (STF, pH 7.4 ± 0.2), good gelling strength ( + + + , gelation in a few seconds, and stability > 24 hours in physiological conditions), and good bioadhesive strength (753.84 dyne/cm^2^). GE-BMopt-IG4 showed extended release of GE up to 12h (78.08 ± 4.73%) than pure GE solution. GE-BMopt-IG4 exhibited significantly higher permeation through excised goat cornea due to the fluidization property of bile salt as well as the bioadhesive characteristic of gelling polymers. GE-BMopt-IG4 did not exhibit any irritation over CAM, excised goat corneas, and restored corneal hydration. GE-BMopt-IG4 also showed a significantly higher anti-microbial effect than pure GE solution due to the sustained release of GE from the GE-BMopt-IG4. The finding revealed that BM-laden *in situ *gel is a good closable alternative carrier of the drug to improve the ocular residence time and therapeutic performance.

## Acknowledgments

 The authors are very thankful to the Deanship of Graduate Studies and Scientific Research at Jouf University for providing the funds for this work.

## Competing Interests

 None.

## Ethical Approval

 Not Applicable.
